# Supplementary material for: Understanding challenges to medical and dental student research practices. An insight from a cross-sectional study of the public sector in Pakistan
Source: PLoS One. 2023 Dec 14;18(12):e0295567. doi: 10.1371/journal.pone.0295567 (PMC10721035; doi:10.1371/journal.pone.0295567)
Supplement: Attachment — Submitted filename: PONE-D-23-18552-Aug 2023.docx [file pone.0295567.s002.docx]

This is an interesting study exploring challenges that keep medical and dental students from public colleges in Pakistan from participating in research opportunities. While the authors raise interesting and important questions, the methods and results of this study are very limited and need significant additional work before the manuscript would be ready for consideration for publication.

Methods:

Authors used a sampling frame of public colleges in a particular region. How many colleges in the catchment area? Public? Private? How many students at those colleges? Those numbers are needed to make sense of the participation.

Need at least 1-2 sentences describing how medical and dental public colleges work in Pakistan – number of years of study; years where medical/dental curriculum is primary, etc.

Survey development – what was covered in the survey? How many questions?

Statistical analysis – was analysis done in excel? Google sheets? Other methods? Need to specify.

Results:

There is very little analysis. Even knowing very little about the survey authors should explore gender differences in responses, differences in year of school, etc. Authors should report participation rates from each school, and give some sense of what proportion of eligible students at each school participated. All of this is necessary to understand the context and to reliably interpret the results of this work.

Writing is rough in parts, and will need additional editing. A few editing suggestions:

L3 ‘dental students enrolled in public sector schools’

L20 ‘including research projects’

L49 ‘participate in research initiatives’

L51 ‘research-centered mindset’

L54 ‘which would encourage students’

L61 ‘Despite an enabling environment’ [here also need to clarify what kinds of research opportunities are available to public college students]

L67 ‘opportunities, and to identify areas for development.’ - I think you mean research opportunities, which is distinct from how someone uses the results of research.

L69 ‘for policy makers’

L73 is Khyber Pakhutunkhwa a Province?

L74 ‘discover the difficulties’

L76 ‘public sector colleges in Pakistan.’ Or ‘public sector colleges in Khyber Pakhutunkhwa.’

L81 ‘the WhatsApp group.’

L86-87 ‘ethical standards. Informed consent was acquired.’

L89 ‘group data in presentation for publication’

L93 ‘dentistry colleges in Khyber Pakhutunkhwa’
