# Supplementary material for: Understanding challenges to medical and dental student research practices. An insight from a cross-sectional study of the public sector in Pakistan
Source: PLoS One. 2023 Dec 14;18(12):e0295567. doi: 10.1371/journal.pone.0295567 (PMC10721035; doi:10.1371/journal.pone.0295567)
Supplement: Attachment — Submitted filename: Rebuttal.docx [file pone.0295567.s003.docx]

Rebuttal

| **Sr. No** | **Comment** | **Rebuttal** |
| --- | --- | --- |
| **1** | “The funders had no role in study design, data collection and analysis, decision to publish, or preparation of the manuscript.”  At this time, please address the following queries:  a) Please clarify the sources of funding (financial or material support) for your study. List the grants or organizations that supported your study, including funding received from your institution.  b) State what role the funders took in the study. If the funders had no role in your study, please state: “The funders had no role in study design, data collection and analysis, decision to publish, or preparation of the manuscript.”  c) If any authors received a salary from any of your funders, please state which authors and which funders.  d) If you did not receive any funding for this study, please state: “The authors received no specific funding for this work.”  Please include your amended statements within your cover letter; we will change the online submission form on your behalf. | The authors did not receive any specific funding or financial/material support for this study. They personally covered all expenses related to data collection, access to relevant literature, analysis, and publication costs. Furthermore, no authors received a salary from external funding sources as there were none involved in this work. |
| 2 | Please amend either the title on the online submission form (via Edit Submission) or the title in the manuscript so that they are identical. | OK, revised accordingly. |
| 3 | Please amend either the abstract on the online submission form (via Edit Submission) or the abstract in the manuscript so that they are identical. | Revised accordingly |
| 4 | Please amend your authorship list in your manuscript file to include author Wajiha Qamar | Revised accordingly to include title page as well with author details |
|  | **Reviewer I** | |
| 1 | Is the manuscript technically sound, and do the data support the conclusions. No | Revised and details added |
| 2 | Has the statistical analysis been performed appropriately and rigorously | Yes |
| 3 | The methods and results of this study are very limited and need significant additional work before the manuscript would be ready for consideration for publication | Details added |
| 4 | Authors used a sampling frame of public colleges in a particular region. How many colleges in the catchment area? Public? Private? How many students at those colleges? Those numbers are needed to make sense of the participation. Need at least 1-2 sentences describing how medical and dental public colleges work in Pakistan – number of years of study; years where medical/dental curriculum is primary, etc. | Information about the number of colleges, the number of students enrolled, and the structure of medical and dental education in Pakistan added. |
| 5 | Survey development – what was covered in the survey? How many questions? | Details added |
| 6 | Statistical analysis – was analysis done in excel? Google sheets? Other methods? Need to specify. | Specified, data entry, cross-checking, and tabulating were done in Excel. |
| 7 | here is very little analysis. Even knowing what we know about the survey authors should explore gender differences in responses, differences in year of school, etc. Authors should report participation rates from each school, and give some sense of what proportion of eligible students at each school participated. All of this is necessary to understand the context and to reliably interpret the results of this work. | Revised |
| 8 | Writing is rough in parts, and will need additional editing. | Revised |
| 9 | Despite an enabling environment’ [here also need to clarify what kinds of research opportunities are available to public college students] | Details added |
| 10 | ‘opportunities, and to identify areas for development.’ - I think you mean research opportunities, which is distinct from how someone uses the results of research. | The author suggests employing the research's findings to identify barriers while offering solutions in order to incorporate the research culture in public sector medical and dental colleges. |
| 11 | is Khyber Pakhutunkhwa a Province? | Yes |
| 12 | public sector colleges in Pakistan.’ Or ‘public sector colleges in Khyber Pakhutunkhwa.’ | Revised |
| 13 | **Reviewer II** | |
| 14 | The research questionnaire has not been shared | Already shared with PLOS One. Re-sharing for convenience |
| 15 | How was the sample stratified? | Details added |
| 16 | Was there any difference between BDS and MBBS students? | While our study initially aimed to compare the experiences of medical and dental students, we ultimately decided to prioritise an understanding of the challenges faced by both groups within the public sector in Pakistan. This approach allowed us to delve deeply into the unique obstacles encountered by each group, ensuring a nuanced exploration of the topic. While we acknowledge the value of potential future research that directly compares these two student cohorts, our study's primary objective was to provide a holistic view of the challenges without compromising the integrity of the data collected. It is pertinent to mention that the study was also not financed by any institution. |
| 17 | **Reviewer III** | |
| 18 | Abstract need to be revised and main objective, method and major findings should be highlighted | Highlighted |
| 19 | LR is quite weak, need to add recent and latest contextual studies. | Revised |
| 20 | Methodology need to justify, why this sample is used? why this analysis approach is adopted? | Justified |
| 21 | Findings need to be reported in a systematic way. | Revised |
| 22 | Discussion is not much clear, it is suggested to support your argument with previous studies. | Revised |
| 23 | Implication of study need to be highlight. | Revised |
